# The SF3b complex: splicing and beyond

**DOI:** 10.1007/s00018-020-03493-z

**Published:** 2020-03-05

**Authors:** Chengfu Sun

**Affiliations:** Non-coding RNA and Drug Discovery Key Laboratory of Sichuan Province, Chengdu Medical College, Chengdu, 610500 China

**Keywords:** Intron, Branch site adenosine (BS-a), Cancer, Modification, Nonsplicing, U2-snRNP-dependent

## Abstract

The SF3b complex is an intrinsic component of the functional U2 small nuclear ribonucleoprotein (snRNP). As U2 snRNP enters nuclear pre-mRNA splicing, SF3b plays key roles in recognizing the branch point sequence (BPS) and facilitating spliceosome assembly and activation. Since the discovery of SF3b, substantial progress has been made in elucidating its molecular mechanism during splicing. In addition, numerous recent studies indicate that SF3b and its components are engaged in various molecular and cellular events that are beyond the canonical role in splicing. This review summarizes the current knowledge on the SF3b complex and highlights its multiple roles in splicing and beyond.

## Introduction

Introns were discovered in the late 1970s [1, 2]. The splicing of introns from nuclear precursors of message RNA (pre-mRNA) is executed by the spliceosome, a ribonucleoprotein (RNP) apparatus that first surfaced in the literature in 1985 [3–5]. Since the discovery of this splicing machinery, characterization of its biochemical composition and catalytic properties has flourished in the splicing field. Early applications of chromatographic fractionation procedures in HeLa cells produced various protein complexes, designated SF factors (SF1 to SF4), that are required for splicing [6]. One of these protein complexes, SF3, was further shown to contain two subcomplexes, SF3a and SF3b [7]. Although the name for SF3b first appeared in 1993, studies on its composition and function, as will be discussed in subsequent sections in this review, were already ongoing at that time. Excellent reviews have recently been published on the structures and functions of U2 small nuclear ribonucleoprotein (snRNP) and SF3b1 in splicing [8, 9]. In this review, I focus on the SF3b complex and discuss its roles in splicing as well as multiple emerging nonsplicing roles.

## Composition and structure

**Fig. 1:**
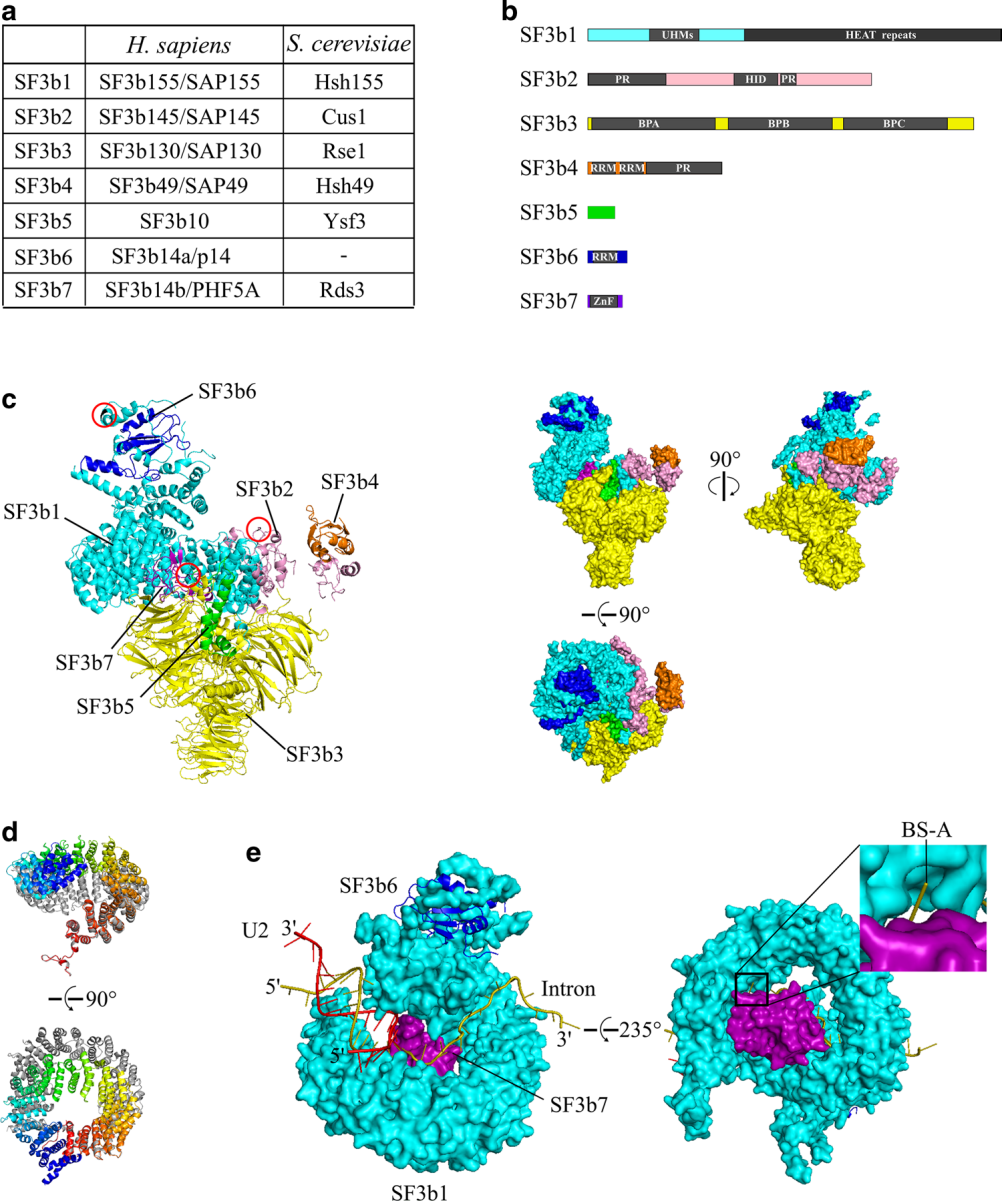
Composition and structure of the SF3b complex. **a** Nomenclature of SF3b components. Names used in human (*H. sapiens*) and budding yeast (*S. cerevisiae*) counterparts are listed for comparison. **b** Schematic domain organization of SF3b components depicted in different colors (SF3b1, cyan; SF3b2, pink; SF3b3, yellow; SF3b4, orange; SF3b5, green; SF3b6, blue; and SF3b7, purple). Domains on each protein are labeled and colored in gray. UHM, U2AF homology motif; HEAT, Huntingtin, EF3, PP2A, and TOR1; PR, proline-rich; HID, HEAT interaction domain; BPA/B/C, β-propeller domain A/B/C; RRM, RNA recognition motif; ZnF, zinc finger. **c** Structure of the SF3b complex. The left panel displays the overall structure of the SF3b complex in cartoon mode with each component labeled. The SF3b complex appears like a flaming torch with SF3b1 on the upper flame part, SF3b3 on the lower torch part and other subunits dotted within or around. The modification-related residues of SF3b1 (Thr434), SF3b2 (Arg508), and SF3b7 (Lys29) are indicated with red circles and colored in black [73, 78, 79]. The right panel shows three views of the SF3b complex in surface mode. The seven SF3b components are colored the same as in **b**. Protein Data Bank (PDB) accession code for the depiction of the structures is 5Z56 [27]. **d** Conformation of the SF3b1 HEAT domain. Superimposition of two conformations of the HEAT domain with the open conformation colored in gray and the closed conformation in rainbow. PDB accession codes for the depiction of the structures are 5Z56 and 5IFE [17, 27]. **e** The structure of SF3b1 binding to U2 and the pre-mRNA intron. Expanded view of the boxed region showing the location of BS-A in a pocket formed by SF3b1 and SF3b7. Proteins are shown in surface mode and RNAs in cartoon. SF3b6, which is distant from BS-A in the structure, is also shown. The pre-mRNA intron is colored in olive and U2 in firebrick. Proteins are colored the same as in **b**, and PDB accession code for the depiction of the structures is the same as in **c**

Characterization of the purified SF3b complex indicated that it consists of seven proteins with a molecular size ranging from 10 to 155 kDa [10–12] (Fig. 1a). Due to methodological differences in identifying SF3b components in human and yeast, a number of names have been designated for these proteins across different species. In this review, I will use SF3b1-7 for consistency and clarity (Fig. 1a).

Seven SF3b components have been shown to contain distinct domains (Fig. 1b). SF3b1 is the largest protein in the SF3b complex, with a notable region containing 20 tandem repeats termed the HEAT domain. This region was originally found in and named after Huntingtin, EF3, PP2A, and TOR1 [13, 14]. In addition, SF3b1 harbors a stretch of U2AF ligand motifs (ULMs) at its N-terminus, which can specifically interact with the U2AF homology motif (UHM) found in proteins, including U2AF65 (also called U2AF2) and Tat-SF1/Cus2 [15]. SF3b2 has no putative structural domains, except for a prominent proline-rich (PR) region at its N-terminus and another, rather short but more conserved, within its C-terminal part [16]. SF3b3 is composed of three seven-bladed β-propeller (BP) domains: BPA, BPB, and BPC. These domains highly resemble the damaged DNA binding protein 1 (DDB1) as well as the cleavage and polyadenylation specificity factor subunit 1 (CPSF1) [10, 17]. On the other hand, SF3b4 contains two N-terminal RNA recognition motifs (RRMs) and a C-terminal PR domain [18]. The remaining three components of the SF3b complex are all small proteins with no structural features in SF3b5, an RRM domain in SF3b6 and a zinc finger (ZnF) domain in SF3b7 [11, 12, 19]. Notably, SF3b5 and/or SF3b6 have been missing from some of the species examined so far.

Recently, atomic structures of the SF3b complex within the spliceosome were resolved [14, 20–28]. The overall architecture of the SF3b complex looks like a flaming torch with SF3b1 and SF3b6 on the upper flame part, SF3b3 on the lower torch part, and the other proteins in between (Fig. 1c). The HEAT domain of SF3b1 is the central scaffold within the complex, which organizes into a superhelical assemblage with structural plasticity and has extensive interactions with other components. SF3b6 is incorporated into the complex by interacting with the N-terminal fragment of SF3b1. SF3b2 contacts the outside of the C-terminal HEAT domain in SF3b1 through a conserved approximately 140-amino acid fragment (herein termed the HEAT interaction domain, HID, see Fig. 1b). This, in turn, links SF3b4 to the middle part of the complex within SF3b. SF3b3 forms the bottom part of the flaming torch and harbors the C-terminal HEAT domain (together with the end anchor region) of SF3b1 and SF3b5 between its BPA and BPC domains. SF3b7, located at the central cavity of the SF3b complex, is surrounded by the circular HEAT domain of SF3b1. In addition to these SF3b cryogenic electron microscopy (cryo-EM) structures within the spliceosome that were already in a U2/branch point sequence (BPS)-bound state, there is also an SF3b crystal structure in its core form, which contains no U2 snRNA or BPS [17]. The SF3b core comprises only the HEAT domain of SF3b1, SF3b3, SF3b5, and SF3b7. A structural comparison between the U2/BPS-bound SF3b and its core suggests that the HEAT domain contains two different conformations: an open conformation in the apo core structure and a closed conformation in the U2/BPS-bound state (see Ref. [8]. for review, Fig. 1d).

## Functional roles in splicing

### Recognizing BPS

The function of SF3b in splicing was initially investigated separately by different groups in the 1990s, and then coalesced into the general view that we currently have. The Reed lab purified spliceosomal complexes from HeLa nuclear extracts and used two-dimensional gel electrophoresis to identify their protein compositions. They subsequently identified a set of spliceosome-associated proteins (SAPs) [29]. Similarly, the Luehrmann lab purified the U2 snRNP from HeLa cells, characterized its composition, and identified several novel proteins that are specific to the slower-sedimenting 17S U2 snRNP [30]. The Krainer lab separated HeLa nuclear extracts into different fractions using chromatographic fractionation and tested their requirements for splicing. They then isolated and named the SF3b complex as mentioned above [7]. The Sharp lab applied site-specifically modified pre-mRNAs for photocrosslinking assays and identified a protein named p14 that specifically recognizes the branch site adenosine (BS-A) during splicing [31]. When all these studies came together, it turned out that some of the SAPs and several novel proteins in the U2 snRNP were the same proteins and that they, together with p14, all belong to the SF3b complex [32, 33]. Finally, the SF3b complex was purified, and all seven components were identified [12]. Identification of homologous proteins of the SF3b complex in other organisms, especially in *Saccharomyces cerevisiae* (except SF3b6), strongly suggests that this multiprotein complex is conserved with evolution.

**Fig. 2:**
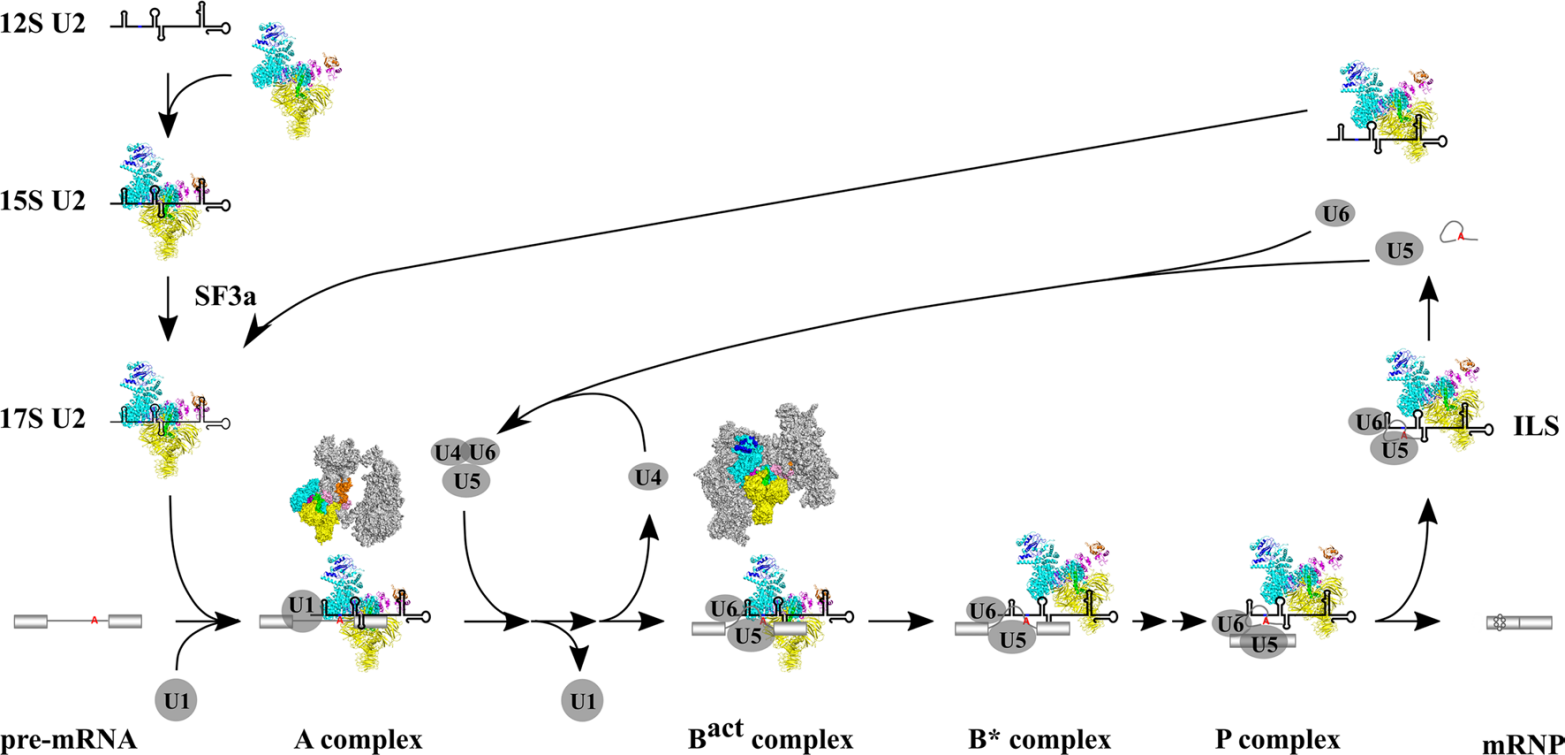
The SF3b complex in splicing. The SF3b complex is first recruited into the U2 snRNP during snRNP maturation, then incorporated into the spliceosome, and binds to BS-A during the spliceosome assembly and activation stages (A to B^act^). Once the spliceosome is catalytically activated (B*), SF3b is displaced from U2/BPS for catalysis to occur and subsequently becomes loosely associated with the spliceosome until the end of splicing [35–40]. Structures of spliceosomal A (PDB accession code: 6G90, [25]) and B^act^ (PDB accession code: 5Z56, [27]) complexes depicted in surface mode and colored in gray are also shown above respective cartoons with SF3b colored the same as in Fig. 1b

Starting from these early works, the basic function of SF3b in splicing was gradually established and has been elaborated with more recent studies (Fig. 2). First, as a component of U2 snRNP, the SF3b complex is recruited to the 12S U2 snRNP, generating the 15S U2 snRNP. Subsequently, another U2 snRNP component, SF3a, is incorporated into the snRNP, forming the functional 17S U2 snRNP [32]. Later, as 17S snRNP enters the spliceosome, the SF3b complex recognizes BS-A in pre-mRNA with its SF3b6 subunit and facilitates an interaction between U2 and BPS [11, 31, 34]. Once catalytic activation of the spliceosome is primed, the SF3b complex is removed from U2/BPS together with several other proteins through the action of the ATPase Prp2 to release the 2′ hydroxyl of BS-A for the subsequent catalysis of transesterification. However, it still binds loosely to U2 within the spliceosome until it finally disassembles from the splicing machinery [35–40] (Fig. 2).

Recent cryo-EM structures of human and yeast spliceosomal complexes (A, pre-B, B, and B^act^) prior to catalytic activation have revealed structural details of SF3b in binding to U2/BPS [14, 20–28] (Fig. 1e). These structures show that the U2/BPS duplex is clamped tightly by terminal HEAT repeats, with the bulged BS-A flipping out of the duplex and being sandwiched in a pocket between the C-terminal HEAT repeats and SF3b7 (hereafter called BS-A pocket, see Fig. 1e). Intron region downstream of BPS runs zigzag on top of the platform formed by the HEAT domain and SF3b7 and exits from the N-terminal HEAT repeats. However, the molecular mechanisms by which SF3b facilitates the formation of U2/BPS during spliceosome assembly are still unclear. In addition, the position of SF3b6, which has previously been shown to biochemically bind to BS-A during splicing, is surprisingly distant from BS-A within the human cryo-EM structures of the spliceosome. This suggests that the recognition of BS-A by SF3b6 may be a much earlier, and presumably transient, event during spliceosome assembly. Interestingly, as SF3b6 is missing in *S. cerevisiae* and seems to also be absent in *Cyanidioschyzon merolae* [41], this event may be different in these organisms.

### Therapeutic drugs affect BPS binding in cancers

The HEAT domain is the central area for RNA and protein binding in the SF3b complex. Its importance is further reinforced by the fact that this domain contains mutation hotspots in several types of cancer, including hematological malignancies and solid tumors [42, 43]. Scrutiny of these cancer-related missense mutations showed that most of them are located in a region that interacts with the intronic sequence downstream of BPS, suggesting that these mutations could affect pre-mRNA binding. Transcriptomic and biochemical studies have shown that these mutations induce the usage of cryptic 3′ splice sites upstream of canonical splice sites, in addition to promoting the selection of alternative branch points, although a structure-guided mutagenesis analysis revealed that mutations on some residues that contact U2 snRNA and encapsulate BS-A appear non-essential and can be tolerated in *S. cerevisiae* [44–48]. In addition, studies in *S. cerevisiae* indicate that the corresponding cancer-related mutations in yeast SF3b1 also alter interactions between SF3b1 and other proteins, including Prp5 and SUGP1, suggesting that mutations in this region have pleiotropic effects in splicing [47, 49, 50].

In addition to these effects of the mutant SF3b1, Obeng and colleagues demonstrated that a spliceosome modulator, E7017, can selectively kill cells expressing SF3b1k700E, a mutant form of SF3b1 [51]. In fact, E7107 is one of the several small molecular splicing inhibitors that specifically target SF3b1, thereby interfering with spliceosome assembly. Interestingly, the cryo-EM structure of the SF3b core bound to E7107 showed that E7107 resides in the BS-A pocket with the HEAT domain of SF3b1 adopting the open conformation [52]. The BS-A pocket likely accommodates other inhibitors that target SF3b1. For instance, pladienolide B, another SF3b1 inhibitor with a distinct structure, was also found to occupy this pocket [53]. These splicing modulators, therefore, inhibit splicing by interfering with the formation of the U2/BPS duplex and blocking the correct BS-A configuration in the spliceosome [54, 55]. Intriguingly, recent studies show that several mutations within the BS-A pocket, either on the HEAT domain of SF3b1 or on SF3b7, confer resistance to these inhibitors [56, 57].

Apart from SF3b1, other SF3b components have also been found to be associated with cancers or genetic diseases. For instance, SF3b2 was reported to positively regulate human prostate cancer and has been implicated to be overexpressed in hepatocellular carcinoma (HCC) [58, 59]. Similarly, SF3b4 has also been implicated in HCC [58, 60–62]. RNA sequencing (RNA-seq) analysis showed that SF3b4 inactivates KLF4, a tumor suppressor, by inducing its aberrant splicing. In addition to HCC, mutations in SF3b4 are involved in the pathogenesis of two types of acrofacial dysostoses: Nager syndrome (NS) and Rodriguez syndrome (RS) [63, 64]. In contrast to SF3b1, mutations resulting from SF3b4 lead to a reduction in its expression levels, which subsequently cause defects in pre-mRNA splicing as well as reduced expression of neural and skeletal genes [65, 66]. SF3b7 is considered a cancer-promoting factor in several types of cancers, including glioblastoma multiforme, lung adenocarcinoma, and HCC [67–71]. RNA-seq analysis showed that alternative splicing patterns are dysregulated in these cancers. Collectively, components of the SF3b complex are deeply involved in various cancers and exert their effects primarily through rewiring the cellular alternative splicing network.

### Modifications of SF3b

The splicing function of the SF3b complex can be regulated and subtly tuned by protein modifications, including phosphorylation/dephosphorylation of SF3b1, methylation of SF3b2 and SF3b4, and acetylation of SF3b7.

The most prominent modification within the SF3b complex is the phosphorylation of SF3b1, which is tightly correlated with the progress of catalysis during splicing [13]. However, the molecular details and biological significance of this modification within the active spliceosome are still poorly understood. To date, two protein kinases that impose phosphate groups on SF3b1 have been identified. Dual-specificity tyrosine-phosphorylation regulated kinase 1A (DYRK1A) is responsible for protein phosphorylation mainly at its Thr434 residue, whereas cyclinE-cdk2 mediates protein phosphorylation within its N-terminus and presumably at multiple sites [72, 73]. Phosphorylated SF3b1 is recognized by nuclear inhibitor of protein phosphatase-1 (NIPP1) and is subsequently directed by the latter to protein phosphatase-1 (PP1) [74, 75]. PP1, together with another phosphatase, PP2A, has been implicated in the progression of the second step of splicing, where it dephosphorylates SF3b1 [76].

In addition to phosphorylation, methylation of the SF3b complex components, including SF3b2 and SF3b4, also affects pre-mRNA splicing [77, 78]. SF3b2 is methylated by PRMT9 at its Arg508 residue, while SF3b4 is methylated by CARM1 within its C-terminal PR region. The methylated SF3b2 is then capable of binding to the Tudor domain of the survival of motor neuron (SMN) protein, suggesting that the methylation of SF3b2 may play a role in coordinating the assembly of U2 snRNP during its maturation. Interestingly, although the U2-related protein SPF30 also contains a Tudor domain, it appears that this domain does not interact with methylated SF3b2 in vitro [78].

Recently, one study reported that SF3b7 is acetylated mainly at its Lys29 residue under nutrient starvation [79]. The study further found that this modification enhances the interaction between SF3b7 and other SF3b components and changes the cellular pre-mRNA splicing patterns. Notably, levels of acetylation in SF3b7 Lys29 are significantly increased in colorectal cancer tissues.

These modification-related residues exhibit a sparse distribution on the structure of the SF3b complex, rendering the acting mechanism of these modifications at a specific splicing stage unlikely (Fig. 1c). Collectively, it is evident that protein modifications on the SF3b complex play multiple roles, including the stabilization of SF3b, biogenesis of U2 snRNP, regulation of pre-mRNA splicing, and progression of tumors.

## Roles beyond splicing

### U2-snRNP-dependent roles of SF3b

#### The 3′-end processing of histone pre-mRNA

**Fig. 3:**
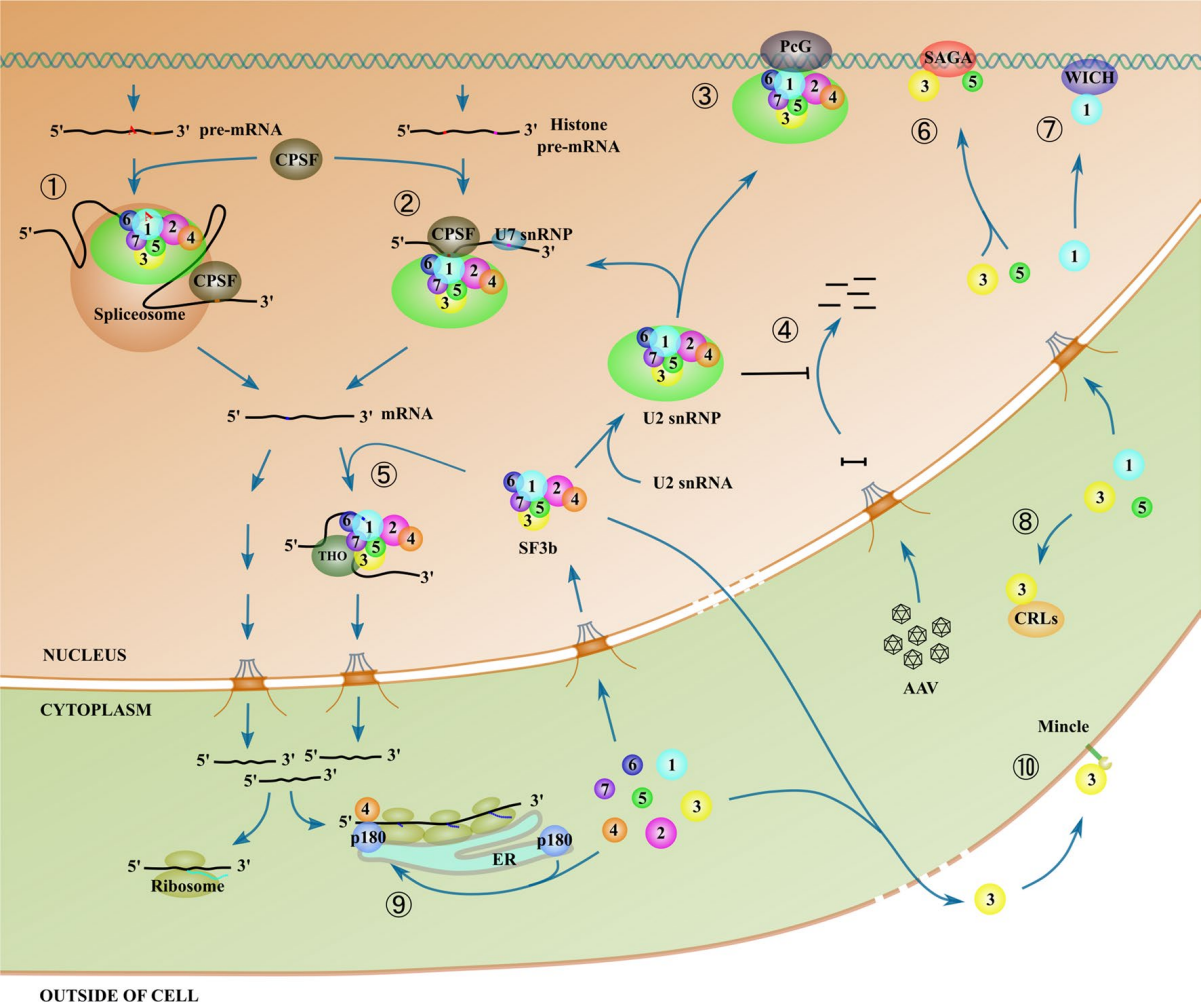
Nonsplicing roles of the SF3b complex. The SF3b complex and its components participate in various molecular and cellular events that are beyond its canonical role in splicing. These events are sorted as either U2-dependent (labeled with circled numbers, ② for the 3′-end processing of histone pre-mRNA [81], ③ for PcG-mediated *Hox* gene repression [84] and ④ for the inhibition of AAV vector transduction [93]) or U2-independent (⑤ for novel mRNA export pathway mediated by THO [83]) for the SF3b complex in the nucleus. SF3b components are also engaged in several biological events that occur in different cellular compartments (⑥ for SF3b3 and SF3b5 containing the SAGA complex [96, 97, 99], ⑦ for SF3b1 containing the B-WICH complex [101], ⑧ for SF3b3 containing CRLs [102, 103], ⑨ for the SF3b4-p180 complex in ER translational control [104], and ⑩ for the SF3b3-Mincle-mediated inflammatory response in necrotic cells [111]). The splicing pathway of the SF3b complex (labeled with ①) is also depicted for completeness and comparison

Nuclear pre-mRNA splicing is not a solitary process but is coupled to other gene expression steps, including transcription, 5′ capping, and 3′-end processing, as well as subsequent nuclear export. For example, a link between splicing and 3′-end processing is achieved by an interaction between U2 snRNP and cleavage and polyadenylation specificity factor (CPSF) (Fig. 3). Kyburz et al. found that U2 snRNP physically interacts with CPSF via their respective subunits, and this interaction is pivotal for the coupling of splicing and 3′-end processing [80]. Notably, this study showed that SF3b3 interacts with its homologous protein CPSF1 (CPSF160 used therein) from the CPSF complex in an in vitro GST pull-down assay. Interestingly, as described below, the interaction between U2 snRNP and CPSF applies well to the processing of histone pre-mRNAs which contain no introns (Fig. 3).

While studying the 3′-end formation of replication-dependent histone pre-mRNAs, Friend et al.found that SF3b1 of the SF3b complex that is within the U2 snRNP contacts, both in vivo and in vitro, a 22-nt RNA element, which is present in the majority of histone pre-mRNAs, in conjunction with a U2 snRNP-related protein Prp43 [81]. Surprisingly, the 22-nt RNA element, especially its most conserved 7-nt motif, has no similarity to the BPS of pre-mRNA introns. It has further been shown that several SF3b components within the U2 snRNP precipitated with the 22-nt RNA element in nuclear extracts, and U2 snRNP also binds to histone pre-mRNAs in *Xenopus laevis* germinal vesicles and HeLa cells, as evidenced by the presence of SF3b1 as well as Prp43 in the respective immunoprecipitation assays; this demonstrates the interaction between this RNA element and the SF3b complex is U2-snRNP-dependent. Previously, SF3b1 was shown to bind to a purine-rich ceramide-responsive RNA cis-element 1 (CRCE1) located in an exon of Bcl-x pre-mRNA [82]. It is unclear how SF3b1, which contains no known sequence-specific RNA binding motifs, can bind this RNA element. Although CRCE1 is comparable in length to the 22-nt RNA element of histone pre-mRNAs, these two elements are different from each other at the sequence level. In addition, SF3b was recently found to be capable of binding to several other sequence motifs, which was examined via SF3b1-enhanced cross-linking and IP (eCLIP) experiments [83]. Taken together, the findings of these studies indicate that SF3b, possibly its SF3b1 subunit, has the ability to bind to many distinct RNA sequences in addition to the canonical BPS in pre-mRNA introns. However, questions remain with regard to how SF3b1/SF3b interacts and whether U2 forms a duplex by base pairing with these RNA sequences.

#### PcG-mediated Hox gene repression

Apart from the aforementioned function of SF3b in the 3′-end processing of histone pre-mRNAs, this complex has also been implicated in the gene repression of *Hox* genes through the action of polycomb group (PcG) proteins (Fig. 3). For instance, Isono et al.demonstrated that the SF3b1 component of the SF3b complex can specifically interact with the Zfp144 and Rnf2 components of polycomb repressive complexes 1 (PRC1), a PcG protein complex [84]. Their interactions were first identified by yeast two-hybrid screening and then confirmed by in vitro glutathione *S*-transferase pull-down and in vivo co-immunoprecipitation (co-IP) assays. The presence of SF3b1 and a U2-specific protein, U2B”, during precipitation with PcG proteins from the nuclear insoluble fraction tentatively suggested that the interaction of SF3b with the PRC1 complex likely occurs in a U2-snRNP-dependent manner. By generating and examining the SF3b1 haploinsufficient mouse models, the authors found that expression levels of SF3b1 were remarkably reduced and the mice exhibited skeletal phenotypes similar to PcG mutants. Notably, decreased levels of SF3b1 led to a dramatic dissociation of SF3b1 with PRC1 proteins, whereas the general splicing patterns seemed unaffected.

Recently, studies have demonstrated that a portion of nuclear U2 snRNPs are either directly or indirectly tethered to chromatin via SF3b or SF3a [85–87]. Conceivably, impeding the association of these U2 snRNPs with chromatin alters pre-mRNA splicing outcomes. In the future, it will be interesting to see what roles other than splicing are played by chromatin-bound U2 snRNPs.

#### Blocking adeno-associated viruses (AAV) vector transduction

RNA splicing is a pivotal step in gene expression, influences many cellular processes, and is also employed by extracellular pathogens, such as viruses. Indeed, the first intron was discovered from adenovirus over 40 years ago. Analyses of the interplay between viral components and cellular splicing factors have shown that many viruses manipulate the cellular splicing machinery in favor of their infections [88]. For example, yeast two-hybrid assays enabled the identification of two components of the SF3b complex, SF3b2 and SF3b7, as interactors with viral proteins, including ICP27 of the herpes simplex virus, Vpr of the human immunodeficiency virus, and NS5A of the swine fever virus [89–92]. Moreover, ICP27 and Vpr block spliceosome assembly and inhibit splicing by hijacking SF3b2.

Recently, Schreiber et al. identified an SF3b component, SF3b7, as a restriction factor for recombinant AAV [93] (Fig. 3). They found that SF3b7, as well as other SF3b proteins and U2AF65 in the U2 snRNP complex, blocks AAV vector transduction in the stage after second-strand synthesis but prior to its transcription. They further showed that cellular restriction of AAV transduction is unlikely due to the effect of recombinant viruses on the cellular splicing machinery but specific to the U2 snRNP complex. This is evidenced by two facts: first, spliceosomal proteins BRR2 and PRRP31, which are the respective U5 and U4/U6 snRNP components of the spliceosome, have no inhibitory effects on AAV vector transduction, and second, the pharmacological inhibition of U2 snRNP by meayamycin B, which targets SF3b1, promotes AAV vector transduction, whereas drugs that modulate other splicing steps show no such effects. In addition, the authors showed that SF3b components, especially SF3b1, interact with the viral capsids after infection. These results indicate that the SF3b complex within U2 snRNP plays an important nonsplicing role in blocking AAV vector transduction.

### The U2-snRNP-independent role of SF3b in mRNA export

As previously mentioned, pre-mRNA splicing is coordinated with other cellular processes during gene expression. After the initiation of RNA transcription and during the splicing stages, two multimeric complexes, the transcription-export (TREX) complex and the exon junction complex (EJC), are gradually deposited on the processing mRNA molecules, and both become engaged in the subsequent mRNA export process. Recently, Wang et al. found that the SF3b complex is involved in TREX recruitment onto both spliced and intronless mRNAs, thereby revealing a novel role of SF3b in RNA export [83] (Fig. 3). The authors showed that components of the subcomplex THO in TREX interact with SF3b components both in vivo and in vitro. Knockdown of SF3b1 resulted in nuclear retention for both spliced and intronless mRNA molecules, whereas tethering the SF3b-binding motif from histone pre-mRNAs (as mentioned above in the “The 3′-end processing of histone pre-mRNA” section) to an exogenous intronless mRNA promoted its export. The study demonstrated that the role played by SF3b here exhibits a U2-snRNP-independent manner, as the disruption of U2 snRNP via an antisense morpholino promoted, instead of inhibited, this SF3b-THO interaction as well as mRNA export. In addition, other U2 snRNP protein components, including SF3a and U2-specific A′ and B″, are dispensable for their interaction. This strongly indicates that the role played by SF3b here is beyond its canonical function in splicing. Apart from the aforementioned findings, three more sequence motifs that SF3b binds to were identified. The ability of SF3b1/SF3b to bind to these heterogeneous sequence motifs opens up new functional possibilities for the SF3b1/SF3b complex/U2 snRNP in RNA metabolism.

A key question to be answered revolves around the source of the SF3b complex for this SF3b–THO interaction. One possible source is the U2 snRNPs in the catalytic spliceosome. Once the spliceosome is primed for catalytic activation, SF3b releases BS-A and repositions itself within the spliceosome during the dramatic rearrangement triggered by the action of Prp2, and then becomes loosely associated with the spliceosome [36, 94]. It is possible that the loosely associated SF3b is subsequently recruited to exons by recognizing its sequence motifs thereon and concomitantly interacts with THO. However, a previous study showed that SF3b components are not significantly present in the spliced mRNPs affinity-purified under physiological conditions from HeLa nuclear extracts. This is although TREX and EJC complexes are both associated with the purified mRNPs, and the substrate pre-mRNA AdML used for this purification contains one of the SF3b recognizing motifs (GAGGA) [95]. It is probable that the coupling between SF3b and THO might be destroyed under the conditions for the purification. Alternatively, another source is the nuclear SF3b pool, where the complex is in a free state without U2 binding. Regarding this possibility, the attendant question is, when and how these SF3b molecules signal the timing for binding to both the sequence motifs on exons and THO proteins.

### Nonsplicing roles of SF3b components

The subsequent sections of this review will discuss the roles of components of the SF3b complex that are outside the range of splicing based on their cellular compartments.

#### SF3b components in the nucleus

Spt-Ada-Gcn5 acetyltransferase (SAGA) is a transcriptional coactivator complex first identified in yeast. A study of human SAGA-like complexes identified SF3b3 as a component in each of the two SAGA complexes, STAGA and TFTC [96, 97]. Although it appears that the protein in SAGA does not affect splicing, its true function in this complex remains elusive. Subsequently, SF3b5 was additionally identified as a SAGA component together with the above SF3b3, based on an interaction with the conserved Sgf29 subunit of the human SAGA complex [98]. Moreover, a recent study showed that SF3b3 and SF3b5 were also found in drosophila SAGA [99]. This suggests that these two proteins may be intrinsic components of SAGA in higher eukaryotes. In this recent study, it was further shown that the expression of a subset of SAGA-regulated genes is affected by disrupting SF3b5 in the fly genome. However, the result is presumably an outcome of the combinational effects of SF3b5’s roles in both splicing and, if it exists, SAGA-related transcriptional activation. In addition to being a component of the SAGA complex, SF3b3 was also found in a nuclear receptor corepressor complex, together with an SF3a protein [100]. In addition, SF3b1 was identified as a component in a high molecular weight chromatin-remodeling complex termed B-WICH [101]. To date, the nature of these interactions remains unclear, while the functions of the SF3b proteins in their respective complexes have yet to be elucidated.

#### SF3b components in the cytoplasm

While studying the regulation of Cullin-RING ubiquitin ligase (CRL) complexes, Menon et al. initially identified SF3b3 as an interacting partner of a subunit from the COP9 signalosome complex. It mediates the deneddylation of Cullins and interacts with several Cullin proteins [102] (Fig. 3). Later, Cordero-Espinoza et al. demonstrated that SF3b3 can negatively regulate the assembly and ligase activity of CRL1 by interfering with the integrity of the complex, whereas it has no influence on other CRLs examined [103]. In the future, studies will be required to explore the molecular aspects of SF3b3 with regard to its role in the ubiquitylation system.

Nascent mRNAs exported from the nucleus must go through ribosomes for translation. When searching for cofactors of p180, an integral endoplasmic reticulum (ER) membrane protein, in ER translational control, Ueno et al. surprisingly found SF3b4 [104]. They showed that SF3b4 interacts specifically with the C-terminal coiled-coil domain of p180. The authors further elaborately demonstrated that SF3b4 cooperates with p180 for efficient protein biosynthesis of COL1A1, the mRNA of the p180-dependent collagen gene, in a manner dependent on a sequence motif located in the 5′ untranslated region (UTR) of COL1A1. This finding clearly provided a unique role for SF3b4 in the translational control on the ER (Fig. 3). In addition, their work provides the possibility that SF3b4, which contains two RRM domains, may preferably bind to some sequence motifs in a direct way, as was first attempted by in vitro RNA selection [105]. The interaction between SF3b4 and the sequence motif identified in this work can easily be examined by a conventional electrophoretic mobility shift assay. Moreover, enhanced cross-linking and IP (eCLIP) can be applied to SF3b4 to explore possible binding motifs, similar to experiments performed by Wang et al. for SF3b1 [83].

A recent study noticed the phenomenon that SF3b1 relocalizes to nuclear and cytoplasmic aggregate foci in response to genotoxic stress in *S. cerevisiae* [106]. It appears that this redistribution pattern is specific to SF3b1, since over 600 other surveyed proteins (including more than a dozen splicing factors) and two SF3b components, SF3b2 and SF3b4, showed no such phenomena. The authors also checked for factors, including intranuclear quality control (INQ) marker proteins and molecular chaperones that influence the formation of aggregate foci of SF3b1. Although subcellular redistribution of nucleus-to-cytoplasm transport under stress conditions has previously been observed in other splicing factors in mammalian and yeast cells, the localization pattern of SF3b1 observed here seems distinct from those factors [107–110]. Nevertheless, further studies are needed to unravel the underlying molecular mechanism of this phenomenon.

#### SF3b components on the cell membrane

In addition to the aforementioned SF3b components that have nonsplicing functions in the nucleus and cytoplasm, one component of this complex, SF3b3, has an extraordinary role: it acts from outside the cell (Fig. 3). While studying the molecular mechanism of the inflammatory response to necrotic cells, Yamasaki and colleagues found that SF3b3 is a ligand of Mincle, a C-type lectin receptor that is expressed on immune cells [111]. Their findings further suggested that upon exposure to various stimuli and stresses such as irradiation, SF3b3 is released from dead cells and then sensed by Mincle via direct binding to its extracellular domain. This process triggers a signal cascade that leads to the production of a series of inflammatory cytokines. Since this discovery, the immunological correlation between SF3b3 and its cognate receptor has been observed under other pathological conditions, including brain and liver injuries [112–115]. However, it is unclear why SF3b3 stands out as the signal molecule for dead cells.

## Conclusions and perspectives

Over 30 years of research on the SF3b complex have established its fundamental function in eukaryotic nuclear pre-mRNA splicing. Its central role in recognizing BPS is embodied, comprehensively, in different molecular and cellular activities, including facilitating spliceosome assembly and activation, regulating cellular alternative splicing networks, and impacting tumorigenesis. In addition to the role played by SF3b in splicing, extensive studies in recent years have gained several novel findings regarding this traditional complex and its components. These findings range from its participation in gene regulation on chromatin to the initiation of cell signaling on the cell membrane. Consequently, a rather integrative view, albeit currently in an incomplete and schematic state, of the multifaceted nature of the SF3b complex should be instituted in our knowledge.

However, basic questions remain to be answered. One open question is the maturation and biogenesis of this multimeric complex. For example, the assembly pathway of the heptameric SF3b complex from its components, as well as its role during U2 snRNP assembly/biogenesis prior to and after splicing in a cellular context, should be investigated. Although cryo-EM structures of the spliceosome prior to catalysis have demonstrated how SF3b interacts with U2/BPS and enwraps BS-A, it remains unclear how the process that SF3b recognizes BS-A and facilitates U2/BPS formation occurring during spliceosome assembly. Furthermore, in comparison to the group II introns, whose BPS is discerned only by RNA elements, there are no clues so far on how such a complicated protein complex as SF3b has evolved for arranging the recognition of the specific RNA sequence during the process of eukaryogenesis. Regarding the emerging nonsplicing roles of the SF3b complex, one intriguing question is how these nonsplicing functions are acquired in higher eukaryotes. Moreover, attention must be paid to carefully discern the diverse functional complexes beyond the spliceosome and whether the isolated SF3b subunit or the holo U2 snRNP/spliceosome is involved. Knowledge of this may lead to a better understanding of the functions of SF3b beyond splicing and its expansion into many other biological processes along with the evolution of organism complexity.
